# Two-Year Efficacy of Cylindrical Annular Refractive Elements Spectacle Lenses for Myopia Control in European Children

**DOI:** 10.1016/j.xops.2026.101309

**Published:** 2026-07-01

**Authors:** Cristina Alvarez-Peregrina, Miguel Angel Sanchez-Tena, Clara Martinez-Perez, Cesar Villa- Collar, Fernandez-Fernandez Victor Ignacio, Nicole Liu, Wayne Li, Padmaja Sankaridurg, Arne Ohlendorf, Clinical Evaluation of MyoCare in Europe (CEME) Study Group, Paula Alves Silva, Diego Asensio Celdrán Vivancos, Raquel Blanco Cotovio, Christina Boeck-Maier, Jose Carlos Garay Dominguez, Ana Isabel Gonzalez Abad, Mariano Gonzalez Perez, Ramon Gutierrez Ortega, Timo Kratzer, Alfredo Lopez Muñoz, Manuel Lérida, Raul Manuel Maia, João Manuel Martinho Antunes, Carolina Mataix Palao, Alejandro Montero Torrejon, Vladimiro Oliveira Hipólito, Jose Ignacio Recalde Zurita, Juan Luis Reina Gallego, Isabel Rodriguez, Alicia Ruiz Hernandez, Antonio Manuel Santos de Melo, Patricia Silva Carrola, Javier Vega Dominguez, Siegfried Wahl

**Affiliations:** 1 Department of Optometry and Vision, Faculty of Optics and Optometry, Universidad Complutense de Madrid, Madrid, Spain; 2 ISEC LISBOA—Instituto Superior de Educação e Ciências, Lisbon, Portugal; 3 Universidad Europea de Madrid, Faculty of Biomedical and Health Sciences, Madrid, Spain; 4 ZEISS Vision Care, Carl Zeiss Vision International GmbH, Aalen, Baden-Württemberg, Germany; 5 ZEISS Vision Care, Carl Zeiss Vision (Guangzhou) Ltd., Guangzhou, China; 6 School of Optometry and Vision Science, University of New South Wales, Sydney, Australia

**Keywords:** Myopia control, Spectacle lenses, Axial length, Pediatric myopia, CARE technology

## Abstract

**Purpose:**

To evaluate the 2-year efficacy of cylindrical annular refractive elements (CARE) spectacle lenses in slowing myopia progression in European children.

**Design:**

Multicenter, double-masked, randomized controlled trial.

**Participants:**

Children aged 6 to 13 years with documented myopia progression of at least < –0.50 diopters (D) in the preceding 12 months.

**Methods:**

Participants were assigned to CARE or single-vision lenses (SVL). Cycloplegic spherical equivalent (SE) and axial length (AL) were measured at baseline, 12, and 24 months.

**Main Outcome Measures:**

Changes in SE and AL over 24 months, and the proportion of rapid progressors (defined as SE change ≤ –0.75 D and AL increase ≥ +0.30 mm over 24 months).

**Results:**

Of the 234 enrolled children, 208 completed the 24-month follow-up. At baseline, mean age was 10.0 ± 1.9 years and mean SE was –2.20 ± 0.94 D. All participants met the inclusion criterion of documented myopia progression of at least –0.50 D in the preceding 12 months. At 12 months, CARE lenses slowed both SE and AL progression compared with SVL (both *P* < 0.001). At 24 months, myopia progression remains lower with CARE than SVL lenses in SE (–0.32 ± 0.20 D vs. –0.69 ± 0.18 D; *P* < 0.001) and AL (0.21 ± 0.11 mm vs. 0.43 ± 0.10 mm; *P* < 0.001). Moreover, the proportion of rapid progressors was lower in the CARE than in the SVL group (30.8% vs. 71.4%; *P* < 0.001). Younger children in the SVL group progressed faster, while CARE lenses mitigated age-related differences.

**Conclusion:**

Cylindrical annular refractive elements lenses effectively slowed myopia progression in European children, with sustained efficacy over 2 years.

**Financial Disclosure(s):**

Proprietary or commercial disclosure may be found in the Footnotes and Disclosures at the end of this article.

Myopia management in children has evolved rapidly over the past decade due to its rising global prevalence and long-term visual risks.^1^ Among available myopia management strategies (optical, pharmacological, light therapies, and environmental approaches), recent evidence syntheses highlight the growing support for optical interventions;^2^, ^3^, ^4^, ^5^, ^6^ spectacle-based approaches are growing rapidly for their ease of use and safety, especially in young children.^7^

Recent advances in spectacle lens designs, involving lens segments or microstructures such as Defocus Incorporated Multiple Segments (Hoya, hoyavision.com),^8^ Highly Aspherical Lenslets (Essilor, essilor.com),^9^ and Diffusion Optics Technology (Sight Glass Vision Inc, sightglassvision.com),^10^ demonstrate higher levels of effectiveness in slowing myopia progression compared to earlier peripheral-plus or progressive addition spectacle lenses.^11^

Similarly, a lens design consisting of cylindrical annular refractive elements (CARE) (MyoCare–CARE, Zeiss Vision Care, zeiss.com) was assessed for its effectiveness in slowing myopia progression in the Asian population.^12^^,^^13^ This lens features a 7-mm central optic zone, intended for refractive error correction, surrounded by a peripheral treatment area composed of multiple concentric rings of microcylinders. The cylindrical annuli are characterized by a radial optical power of +9.2 diopters (D) and no power (0.0 D) in the circumferential axis, resulting in a mean cylindrical power of +4.6 D (Fig 1). The alternating pattern of cylindrical and clear zones is designed to induce simultaneous defocus in the peripheral retina, a mechanism thought to play a key role in slowing myopia progression.^14^ After 2 years of lens wear, the results were favorable with CARE and CARE S spectacles demonstrating significant slowing of myopia in Asian children.^13^

**Figure 1:**
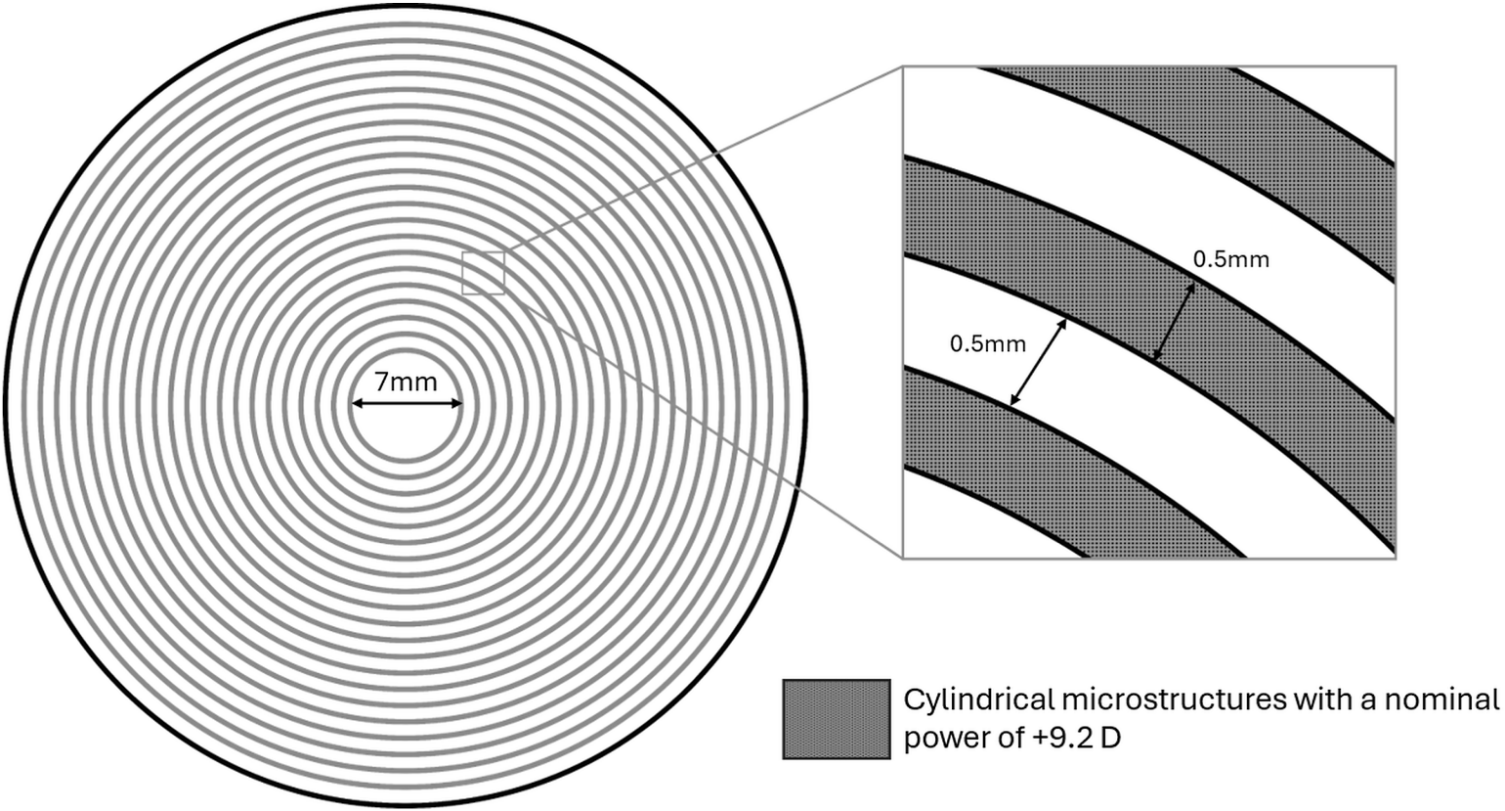
Schematic overview of the cylindrical annular refractive elements in the CARE lens design. CARE = cylindrical annular refractive elements; D = diopters.

Most research on myopia management has been conducted in Asia, particularly among Chinese children, where prevalence is the highest.^3^^,^^15^ As genetic and environmental factors differ across regions,^16^ the applicability of these findings to European cohorts remains uncertain.^2^ In this context, previous findings from the Clinical Evaluation of MyoCare in Europe trial demonstrated that CARE lenses significantly slowed myopia progression in European children after 1 year of follow-up.^17^ Building on these results, the present study evaluates the durability of the treatment beyond the first year.^18^ This study extends those findings by evaluating the durability of the treatment over 2 years.

## Methods

The Clinical Evaluation of MyoCare in Europe study is an ongoing, prospective clinical trial with a randomized, controlled, and double-masked design conducted across 6 ophthalmic clinics in Spain and Portugal: Novovision (Madrid, Spain), Instituto Clínico Quirúrgico de Oftalmología (Bilbao, Spain), Clínica Privada de Oftalmología (Lisbon, Portugal), Miranza (Seville, Spain), Novovision (Murcia, Spain), and Instituto de Microcirugía Ocular (Madrid, Spain). It was registered at clinicaltrials.gov under the code NCT05919654. Ethical approval was granted by the Ethics Committee of Hospital Clínico San Carlos, and written informed consent was obtained from all parents or legal guardians, together with an assent form from children aged ≥12 years. All procedures adhered to the principles of the Declaration of Helsinki for research involving human participants.

### Trial Interventions

A total of 318 children were screened for eligibility. Inclusion criteria were age 6 to 13 years, European ancestry, myopia between –0.75 and –5.00 D (cycloplegic spherical equivalent [SE]), best-corrected visual acuity ≤0.00 logarithm of the minimum angle of resolution (monocular and binocular), cylinder ≤ –1.50 D, anisometropia ≤ –1.00 D, and documented myopia progression ≤ –0.50 D in the previous year. Exclusion criteria included ocular pathology, prior ocular surgery, prior myopia management treatments, and contraindications to cycloplegic or anesthetic eye drops. Table 1 shows the assessments performed at each visit. Full details on study design, recruitment, randomization, and outcome measures have been published elsewhere.^19^

**Table 1:** Schedule of Study Visits and Assessments Performed

Visit	VA	Subj Rx	Cycloplegic Rx	AL	Questionnaires	Notes
Baseline	X		X	X	X∗	Initial measurement
Visit 0	X					Spectacle dispensing
Visit 1 (1 wk)	X			X	X†	1-week follow-up
Visit 2 (3 mos)	X			X	X†	3-month follow-up
Visit 3 (6 mos)	X	X	X	X		6-month follow-up
Visit 4 (12 mos)	X	X	X	X		12-month follow-up
Visit 5 (18 mos)	X	X	X	X	X‡	18-month follow-up
Visit 6 (24 mos)	X	X	X	X	X∗‡	24-month follow-up

AL = axial length; Rx = refraction; VA = visual acuity.

∗ Environmental and genetic factors questionnaire.

† Telephone questionnaire regarding wearability.

‡ Adherence questionnaire.

After screening, 234 participants were enrolled and randomly allocated to either the single-vision lenses (SVL) or CARE lenses (MyoCare, ZEISS Vision Care, zeiss.com). Spectacles were fitted following manufacturer guidelines to ensure optimal positioning and visual performance. Of these, 226 completed the first year and continued into year 2, with follow-up visits at 18 (Visit 5) and 24 months (Visit 6).

These visits included evaluation of visual acuity, subjective refraction, cycloplegic autorefraction, and axial length (AL). Visual acuity was assessed at each visit, both monocularly and binocularly, and with and without correction. Lenses were replaced if visual acuity dropped by one line or SE changed by ≤ –0.50 D. Cycloplegic refraction was measured using a wavefront-based autorefractor (i. Profiler; Carl Zeiss Vision, zeiss.com) 30 minutes after 2 instillations of 1% tropicamide. Spherical equivalent was calculated as the sum of the sphere and the half-cylinder. The mean of 3 consecutive measurements was used as the final value. Axial length was determined as the average of 5 measurements obtained with the IOLMaster 700 biometer (Carl Zeiss Meditec AG, Jena, zeiss.com). Lifestyle questionnaires captured parental myopia, outdoor time, near-work activities, and screen use. All procedures performed during these visits were consistent with those outlined in the initial study protocol, ensuring continuity and standardization of data collection. Children were instructed to wear spectacles full-time, and compliance was assessed at 18 and 24 months via self-reported questionnaires. Daily use ≥10 hours per day was considered adequate, and questionnaires also evaluated lens wear during near-based activities.

### Sample Size

The required sample size was determined based on published estimates of annual myopia progression in European children wearing SVL and the expected treatment effect of CARE lenses. An average progression of approximately –0.50 D per year in the control group and a 50% reduction with the intervention were assumed. Under these assumptions, 106 participants per arm were estimated to provide 80% power to detect this difference at a 2-sided α level of 0.05. A standard deviation of 0.65 D for annual refractive change, consistent with previous reports, was incorporated into the calculation. To account for an anticipated dropout rate of around 10% over the study period, the target enrollment was increased to 234 children.

### Statistical Analysis

Descriptive statistics were used to summarize baseline characteristics and progression outcomes. Myopia progression was assessed as the absolute change in SE and AL from baseline to 24 months. Children with SE change ≤ –0.75 D and AL increase ≥ +0.30 mm over 24 months were classified as rapid progressors.

Group comparisons between the CARE and SVL groups were performed using independent *t* tests and χ^2^ tests. Analyses of compliance were conducted exclusively within the CARE group, as compliance does not apply to SVL. The predefined compliance threshold was ≥10 hours/day, and an additional exploratory analysis was performed using ≥12 hours/day, consistent with previous myopia-control trials.

Multivariate linear regression models were used to assess the association between compliance and SE/AL progression, adjusting for baseline SE or AL, age, gender, parental myopia, and recruitment center. Logistic regression estimated the odds of rapid progression by treatment group and compliance status. Analyses were conducted using Stata v17.0 (StataCorp), with significance set at *P* < 0.05.

In addition to the primary analyses, several prespecified complementary analyses were conducted: (1) evaluation of SE and AL at 6-month intervals, (2) age-stratified analyses (6–9 vs. 10–13 years), (3) comparison of year 1 versus year 2 progression, and (4) contextualization of axial elongation using the meta-regression model proposed by Brennan et al.^20^ This model was not used for imputation of missing data, but rather to estimate expected physiological axial elongation in untreated European children based on age and race. The formula applied was:

Ln(AxialElongation)=0.362–0.158(Age+0.5)+0.325(RaceCode)

Race code of 0 was used because of the European ancestry of the subjects. Age was computed for each visit using the child’s exact date of birth and the visit date, meaning that the mid-year correction recommended by Brennan et al was inherently accounted for. Expected AL elongation was calculated for each child and compared with observed elongation in the CARE group at 12 and 24 months to contextualize the treatment effect relative to physiological growth.

## Results

### Participant Flow and Baseline Characteristics

**Figure 2:**
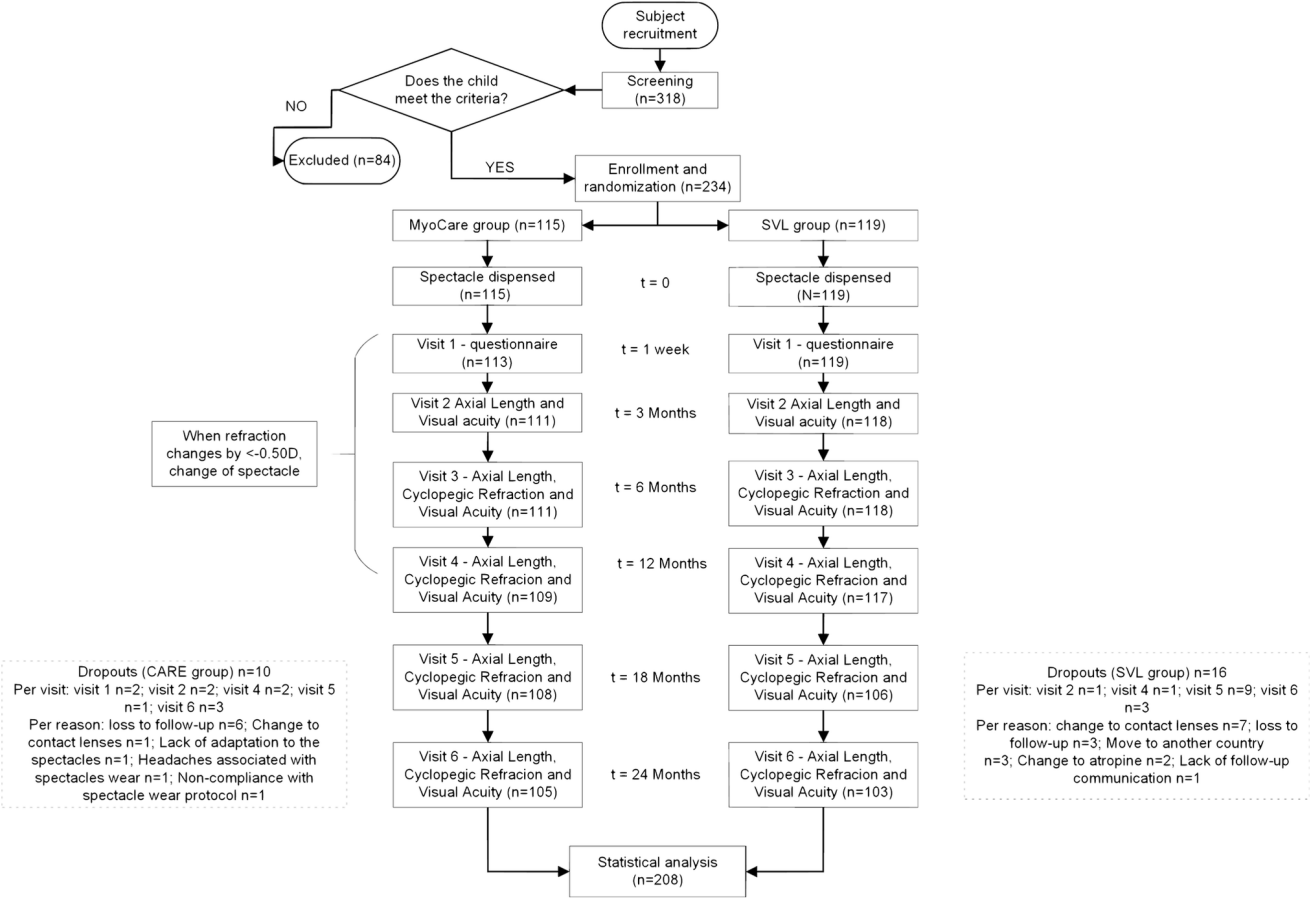
Study flowchart. CARE = cylindrical annular refractive elements; D = diopter; SVL = single-vision lenses.

Of the 234 children enrolled, 226 completed the 12-month visit. At 24 months, data from 208 children were available (CARE: n = 105; SVL: n = 103), with one excluded from AL analyses due to missing data. From 12 to 24 months, there were 4 dropouts in the CARE group and 14 in the SVL group, mainly due to relocation, lack of adaptation, treatment switch, and loss to follow-up. Baseline demographic, refractive, and behavioral characteristics were comparable between groups, including age, SE, AL, parental myopia, outdoor time, and near-work patterns (Table 2). Figure 2 shows the study flowchart and visit completion.

**Table 2:** Baseline Demographic, Refractive, Familial, and Behavioral Characteristics of Participants by Treatment Group

Category	CARE (n = 115)	SVL (n = 119)	*P* Value
Demographics			
Age (yrs), mean ± SD	10.3 ± 1.9	9.8 ± 1.9	0.077
Female (%)	55.70%	56.30%	1.000
Refractive status			
Spherical equivalent (D), mean ± SD	–2.28 ± 0.94	–2.12 ± 0.94	0.189
Axial length (mm), mean ± SD	24.34 ± 0.68	24.17 ± 0.75	0.075
Family history			
≥1 myopic parent (%)	79.00%	79.00%	0.999
Behavioral factors			
Outdoor time <1.6 hrs/day (%)	45.60%	47.00%	0.843
Near-work >3 hrs/day (%)	36.90%	33.60%	0.852
Digital device use >50% of near-work (%)	35.10%	38.60%	0.767

CARE = cylindrical annular refractive elements; D = diopters; SD = standard deviation; SVL = single-vision lenses.

### Myopia Progression from Baseline to 24 Months

Myopia progression over 24 months was significantly slower in children wearing CARE lenses compared to SVL. The observed mean SE change was –0.70 ± 0.55 D in SVL versus –0.31 ± 0.46 D in CARE; AL change was 0.43 ± 0.26 mm versus 0.21 ± 0.20 mm, respectively. The overall mean differences of 0.39 D and 0.22 mm with slower progression in CARE were statistically significant (*P* < 0.001 for both).

**Figure 3:**
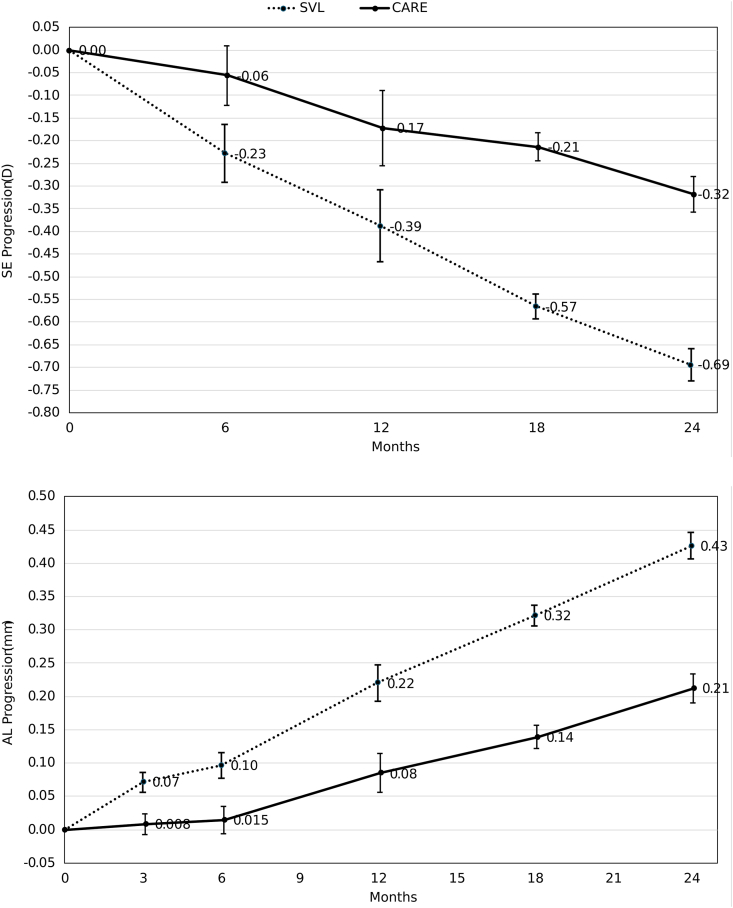
Adjusted change in (**A**) SE and (**B**) AL over time. ∗Adjusted mean, bar representing 95% CI. AL = axial length; CARE = cylindrical annular refractive elements; CI = confidence interval; D = diopter; SE = spherical equivalent; SVL = single-vision lenses.

After adjusting for age, gender, baseline values, parental myopia, and center, SE change was –0.69 D (95% confidence interval [CI]: –0.73 to 0.66) in SVL versus –0.32 D (95% CI: –0.36 to 0.28) in CARE (mean difference of 0.38 D, *P* < 0.001). Adjusted AL changes were 0.43 mm (95% CI: 0.41‑0.45) in SVL versus 0.21 mm (95% CI: 0.19‑0.23) in CARE (mean difference of 0.22 mm, *P* < 0.001), with differences between groups evident at each 6-month interval (Fig 3).^20^

Supplementary Table 1 (available at www.ophthalmologyscience.org) shows the details of the observed, adjusted, and calculated changes.

### Rapid Progressors with Single Vision and CARE Lenses

Rapid progression was significantly less frequent in the CARE group. Based on SE change, 15.4% of CARE participants met the rapid progression threshold versus 42.2% in SVL (χ^2^ = 18.06, *P* < 0.001); for AL, 26.0% versus 69.6% (χ^2^ = 39.33, *P* < 0.001). Using either criterion, 27.9% in CARE were classified as rapid progressors compared to 69.6% in SVL (χ^2^ = 35.89, *P* < 0.001). Finally, when considering both criteria, 13.5% of CARE participants were classified as rapid progressors compared to 42.2% in SVL (χ^2^ = 21.19, *P* < 0.001). These results are presented in Table 3.

**Table 3:** Summary of the Main Observed Results of the 24-Month Analysis of CARE Efficacy

Category	Subgroup	SE Change (D)	AL Change (mm)	Fast Prog. (%)∗	*P* Value (SE)	*P* Value (AL)	*P* Value (FP)
		Mean ± SD	Mean ± SD				
Treatment vs. control	SVL	–0.70 ± 0.55	0.43 ± 0.25	36.1			
	CARE	–0.31 ± 0.46	0.21 ± 0.20	12.2			
	Difference	0.39	–0.22	–24.00	< 0.001	< 0.001	< 0.001
Age group 6-9 yrs	SVL	–0.83 ± 0.56	0.54 ± 0.27	49.1			
	CARE	–0.38 ± 0.47	0.28 ± 0.20	13.3			
	Difference	0.46	–0.26	–35.7	0.001	0.001	< 0.001
Age group 10-13 yrs	SVL	–0.59 ± 0.52	0.33 ± 0.20	25.8			
	CARE	–0.26 ± 0.44	0.17 ± 0.19	11.4			
	Difference	0.32	–0.16	–14.3	0.004	0.003	< 0.001

AL = axial length; CARE = cylindrical annular refractive elements; D = diopters; FP = fast progressors; SD = standard deviation; SE = spherical equivalent; SVL = single-vision lenses.

∗ FP: fast progressors who met both criteria (AL change ≥0.30 mm and SE change ≤ –0.75 D).

To ensure these findings were not influenced by baseline imbalances, a subanalysis was conducted comparing the initial characteristics of rapid progressors in both groups. No significant differences were observed between CARE and SVL rapid progressors in terms of baseline age (9.65 ± 1.93 vs. 9.34 ± 1.80 years; *P* = 0.562), SE (–2.31 ± 0.79 vs. –2.27 ± 1.03 D; *P* = 0.886), or AL (24.30 ± 0.71 vs. 24.09 ± 0.81 mm; *P* = 0.374). Furthermore, lifestyle factors (outdoor time and near work) and parental myopia history were also well balanced between these subgroups (all *P* > 0.05), as detailed in Supplementary Table 2 (available at www.ophthalmologyscience.org). These results confirm that the groups were comparable at inclusion and that the observed differences in progression rates are attributable to the treatment.

Among rapid progressors, SE and AL changes were slightly lower in CARE compared with SVL (SE: –0.85 ± 0.33 D vs. –0.93 ± 0.49 D; AL: 0.46 ± 0.14 mm vs. 0.55 ± 0.21 mm). The difference in SE was not statistically significant (*P* = 0.227), whereas AL change showed a significant reduction in the CARE group (*P* = 0.042). Among nonrapid progressors, myopia progression was minimal in both groups but consistently lower in CARE. Spherical equivalent change was –0.09 ± 0.28 D in CARE versus –0.18 ± 0.25 D in SVL, with no statistically significant difference between groups (*P* = 0.062). In contrast, AL change showed a significant reduction in the CARE group (0.11 ± 0.12 mm) compared with SVL (0.16 ± 0.09 mm; *P* = 0.018).

### Compliance and Treatment Efficacy

At 24 months, spectacle wear time was comparable between groups. Using a threshold of ≥12 hours/day, 82.8% of children in the CARE group and 87.8% of those in the SVL group met the criterion, with no statistically significant differences in adherence between study arms (*P* = 0.322). Ten participants across both groups were excluded from this analysis due to missing adherence data.

Within the CARE group, no significant differences in SE or AL progression were found between compliant and noncompliant groups, whether using a ≥10 hours/day or a stricter ≥12 hours/day threshold. Furthermore, subanalyses demonstrated that CARE lenses remained significantly more effective than SVL lenses even among participants with lower compliance (<12 hours/day) for both SE (*P* = 0.012) and AL (*P* = 0.005). Additionally, 18 children (17.1%) in the CARE group reported removing their spectacles during near tasks, but this did not impact treatment outcomes.

### Age-Based Efficacy Analysis

Analysis by age group showed that younger children (aged 6–9 years) in the SVL group had significantly greater SE progression (–0.83 ± 0.56 D) than older children (–0.59 ± 0.52 D, *P* = 0.020). In contrast, no significant differences were found between age groups in the CARE group (SE change –0.38 ± 0.47 D in younger children and –0.26 ± 0.44 D in older ones, *P* = 0.285).

Similarly, younger children on SVL had greater axial elongation (0.54 ± 0.27 mm) than older children (0.33 ± 0.20 mm, *P* < 0.001), while with CARE, the difference was smaller (0.28 ± 0.20 mm vs. 0.17 ± 0.19 mm, *P* = 0.004). In summary, overall younger children progressed more and CARE lenses reduced this age-related disparity.

Rapid progression was more common in younger SVL children (49.1% vs. 25.8%, *P* = 0.009), but not significantly different by age in CARE (13.3% vs. 11.4%, *P* = 0.760). Table 3 shows a summary of the observed results previously described.

### Year 1 and Year 2 Change in SE and AL

Cylindrical annular refractive elements lenses consistently reduced myopia progression across both years. In year 1, axial elongation was 0.09 ± 0.14 mm in CARE versus 0.23 ± 0.15 mm in SVL (*P* < 0.001); in year 2, 0.11 ± 0.09 mm versus 0.19 ± 0.16 mm (*P* < 0.001), with mean differences of 0.14 mm and 0.08 mm, respectively.

These values correspond to annualized treatment efficacy of 0.14 mm in year 1 and 0.08 mm in year 2, which highlights the stronger effect during the first year and the sustained, although slightly reduced, effect during the second year.

Spherical equivalent progression followed a similar pattern: –0.20 ± 0.41 D versus –0.41 ± 0.41 D in year 1 and –0.12 ± 0.32 D versus –0.28 ± 0.36 D in year 2 (*P* < 0.001 for both) with CARE versus SVL, respectively.

## Discussion

The 2-year results from this ongoing multicenter trial confirm the sustained efficacy of CARE lenses in slowing myopia progression in European children. The results build upon the first-year findings of the Clinical Evaluation of MyoCare in Europe study, which showed a significant reduction in SE and AL after 12 months of lens wear.^17^ Importantly, the treatment effect persisted into the second year, although the magnitude of change was slightly attenuated. These results demonstrate that CARE lenses maintain efficacy over time, with consistent benefits across both years of treatment.

In our European cohort, adjusted mean SE change over 24 months was –0.32 D in CARE versus –0.69 D in SVL, and AL elongation was 0.21 mm versus 0.43 mm. These results align with the recent Asian CARE trial, where Chinese children wearing CARE lenses showed a 2-year SE progression of –0.73 D and AL elongation of 0.40 mm, compared with –1.15 D and 0.59 mm in SVL. The absolute treatment effect in both studies (0.44 D in SE and 0.20 mm in AL) was remarkably similar despite differences in baseline progression rates and lifestyle.^13^ While our findings mirror those in Asian cohorts, it is important to address the higher dropout rate observed in our SVL group. This led to a baseline imbalance in AL among those who completed 24 months, with the CARE group exhibiting a slightly higher baseline AL. To ensure this did not bias our efficacy results, an analysis of covariance was performed to adjust the 24-month axial elongation by baseline AL. The analysis confirmed that initial eye size did not significantly influence the elongation rate in our model (*P* = 0.081). Furthermore, after adjusting for baseline AL, the treatment effect remained highly significant (*P* < 0.001), with an adjusted mean elongation of 0.213 mm for the CARE group compared with 0.421 mm for the SVL group (mean difference 0.208 mm). This indicates that the efficacy of the CARE optical design is robust and independent of the initial axial dimensions of the eye.

Additionally, we conducted an additional analysis using the Brennan et al meta-regression model,^20^ which estimates age expected emmetropic AL based on age and race. An increasing number of recent studies have applied this formula to account for the absence of a randomized single-vision group.^21^, ^22^, ^23^

For European ancestry, expected AL growth was calculated and compared to observed elongation in CARE wearers. Based on age and baseline AL, the expected change over 12 and 24 months was 0.27 mm and 0.50 mm, respectively. These estimates were higher than the observed SVL rates (0.22 mm and 0.43 mm), resulting in a difference of 0.18 mm and 0.29 mm between the untreated and CARE groups—greater than the observed difference of 0.14 mm and 0.21 mm. As the Brennan model aggregates data from multiple non-Asian populations, it provides a global age-specific reference rather than a population-specific benchmark. Therefore, differences between predicted and observed growth should be interpreted as contextual indicators rather than direct estimates of treatment effect.

The 24-month treatment effect observed in our study is clinically competitive when compared with other established optical designs. In European cohorts, Defocus Incorporated Multiple Segments lenses have shown an axial elongation of 0.26 mm over 2 years,^23^ which is closely aligned with the 0.21 mm observed in our CARE group. Furthermore, our 2-year efficacy (0.21 mm) compares favorably with the 0.21 mm effect reported for Diffusion Optics Technology in North American and European “full-time wearers.”^24^ While technologies tested in Asia, such as Highly Aspherical Lenslet, often report larger absolute effects (e.g., 0.41 mm in 2 years),^25^ these differences are primarily driven by the higher baseline progression rates of Asian children. When normalized for population-specific progression, CARE lenses demonstrate a robust performance profile consistent with current gold-standard treatments.

Age at treatment initiation emerged as a key factor in progression. In the SVL group, younger children (aged 6–9 years) progressed significantly faster than older children (aged 10–13 years), both in SE and AL. These results suggest that younger children tend to progress faster, especially in the absence of treatment.^26^ Our analysis confirmed that age is a significant independent factor in myopia progression, showing a stronger effect on axial elongation (*P* < 0.001) than on refractive change (*P* = 0.008). Younger children (aged 6–9 years) exhibited higher progression rates in both parameters. However, formal interaction analyses for both SE (*P* = 0.350) and AL (*P* = 0.104) revealed that the treatment effect of CARE lenses is consistent across age groups. Therefore, while the lenses significantly mitigate progression in all children, they do not fully override the inherent biological differences related to age; younger participants continue to exhibit higher absolute progression than older ones, albeit at a reduced rate compared with the control group. These findings are consistent with previous reports indicating that younger age is associated with faster myopia progression and that earlier onset leads to higher ultimate levels of myopia and an increased risk of developing pathological myopia.^27^^,^^28^ The ability of CARE lenses to significantly slow progression in these high-risk younger children is encouraging for their clinical use.

Compliance is another critical factor influencing treatment efficacy.^29^ In our cohort, spectacle wear time was high and comparable between groups; 82.8% of the CARE group and 87.8% of the SVL group used their lenses for >12 hours daily (*P* = 0.322), indicating that adherence was balanced across study arms. This high level of compliance is consistent with reports from other leading technologies where wear time is key to maximizing treatment effect. For example, in studies involving Highly Aspherical Lenslet and Diffusion Optics Technology lenses, optimal results were more frequently observed in “full-time wearers” or those with wear times exceeding 12 to 14 hours.^10^^,^^25^ While our data did not show significant differences between high and low compliance groups, the overall treatment effect remained robust. Notably, subanalyses demonstrated that CARE lenses remained significantly more effective than SVL even among participants with lower compliance (<12 hours/day) for both SE (*P* = 0.012) and AL (*P* = 0.005). However, it is important to acknowledge that the high overall adherence (>82% wearing spectacles ≥12 hours/day) resulted in a relatively small noncompliant subgroup. This lack of a larger range in wear time may have limited our ability to identify a significant dose-response relationship, and the absence of differences between these groups should be interpreted with caution due to the limited statistical power of this specific comparison. Nevertheless, the consistent performance of the CARE lens across different wear-time profiles highlights its robustness in clinical practice.

Rapid progressors represent a subgroup of particular clinical interest. In our study, the proportion of children meeting the defined rapid progression criteria (SE change ≥ –0.75 D and AL elongation ≥ +0.30 mm) was significantly lower in the CARE group compared to SVL. Moreover, among rapid progressors, the magnitude of progression was reduced in the CARE group. Specifically, even within this high-risk subgroup, children wearing CARE lenses showed significantly lower mean changes in AL compared with those wearing SVL lenses, indicating that the treatment effect persists even in those most prone to progression. The baseline comparability of the rapid-progressor subsets in both arms (as shown in Supplementary Table 2) strengthens the conclusion that the CARE lens effectively alternates the progression trajectory in children who would otherwise experience fast myopic changes. These findings are consistent with the Asian CARE trial, where survival analysis showed that 75% of CARE wearers avoided progression of ≥1.25 D over 2 years, compared with only 50% in SVL.^13^ This suggests that CARE lenses can be very beneficial for children at higher risk of developing high myopia and its associated complications.

The durability of the treatment effect is also noteworthy.^30^ As observed in other trials, the efficacy of myopia management interventions is typically strongest during the first year and then modestly reduced in the second year. In our study, this pattern was clearly reflected in the annualized axial elongation differences between CARE and SVL, which were 0.14 mm in year 1 and 0.08 mm in year 2. Reporting annualized efficacy follows the recommendations by Flitcroft^31^ for interpreting and designing future myopia-control trials, as year-by-year estimates provide a more transparent understanding of treatment performance over time. Despite the expected attenuation, the reduction in SE and AL remained significant at 24 months. This pattern was also evident in the Asian CARE trial, where the first-year SE reduction was 0.37 D and the second-year reduction was 0.15 D. Although the effect was slightly attenuated in the second year, CARE lenses remained effective in slowing myopia, a pattern consistent with other optical interventions where the first-year effect is typically stronger.

Taken together, our findings confirm that CARE lenses demonstrated a favorable safety profile and high clinical efficacy for myopia management in European children. Importantly, the efficacy of the lens in this group of European children was like that observed in Asian populations and indicates that race may not be a factor that influences the efficacy of these lenses. The treatment effect is sustained over 2 years, with particular benefit in younger children and rapid progressors. These results support the inclusion of CARE lenses in clinical protocols for early myopia management and highlight the importance of timely intervention to reduce long-term visual morbidity.

## Conclusion

Cylindrical annular refractive elements spectacle lenses demonstrated a favorable safety profile and a high clinical efficacy option for myopia management in European children. Their sustained efficacy over 2 years, particularly in younger and rapid-progressing children, supports their inclusion in early intervention protocols. These findings contribute to the global evidence base for optical myopia management and inform clinical practice across diverse populations.
