# Supplementary material for: Two-Year Efficacy of Cylindrical Annular Refractive Elements Spectacle Lenses for Myopia Control in European Children
Source: Ophthalmol Sci. 2026 Jul 1;6(9):101309. doi: 10.1016/j.xops.2026.101309 (PMC13452212; doi:10.1016/j.xops.2026.101309)
Supplement: Supplementary file 1 [file mmc1.pdf]

Supplementary Table 1. Baseline characteristics of matched participants

Variable	MCI group (n=387)	Non-MCI group (n=387)	Standardized Mean Difference
Age, years	71.9±5.2	71.4±4.9	0.057
Gender (female), no. (%)	241 (62.3)	255 (65.9)	0.036
Education years, years	12.0±2.5	12.0±2.1	0.072
Household incomes, million yen			0.057
0 – 1.99	93	71	
2.00 – 3.99	186	205	
4.00 – 5.99	58	72	
6.00 – 7.99	26	18	
8.00 – 9.99	13	10	
>10	11	11	
Diabetes mellitus, no. (%)	32 (8.2)	37 (9.6)	0.013

Data are given as mean ± standard deviation unless otherwise indicated.
