# Supplementary material for: Two-Year Efficacy of Cylindrical Annular Refractive Elements Spectacle Lenses for Myopia Control in European Children
Source: Ophthalmol Sci. 2026 Jul 1;6(9):101309. doi: 10.1016/j.xops.2026.101309 (PMC13452212; doi:10.1016/j.xops.2026.101309)
Supplement: Supplementary file 2 [file mmc2.pdf]

Supplementary Table 2. Multivariable analysis of associations of saccadic eye movement parameters with the presence of mild cognitive impairment

Varible	Odds	95% CI	*P* value
Reaction time (/100msec)	1.22	1.11 to 1.36	**<0.001**
Latency (/100msec)	1.33	1.01 to 1.76	**0.045**
No. of saccade	1.22	1.00 to 1.51	0.051
First gain	0.31	0.10 to 1.00	0.051
End gain	0.21	0.04 to 0.96	**0.048**

CI = confidence interval; msec = milliseconds.
